# Supplementary figures and images for: Distinct and combined interferon-ɑ/β-receptor-1 loss in neurons and astrocytes disrupt brain energy metabolism and drive Parkinsonian dementia
Source: J Biomed Sci. 2026 Jun 1;33:57. doi: 10.1186/s12929-026-01257-8 (PMC13227799; doi:10.1186/s12929-026-01257-8)

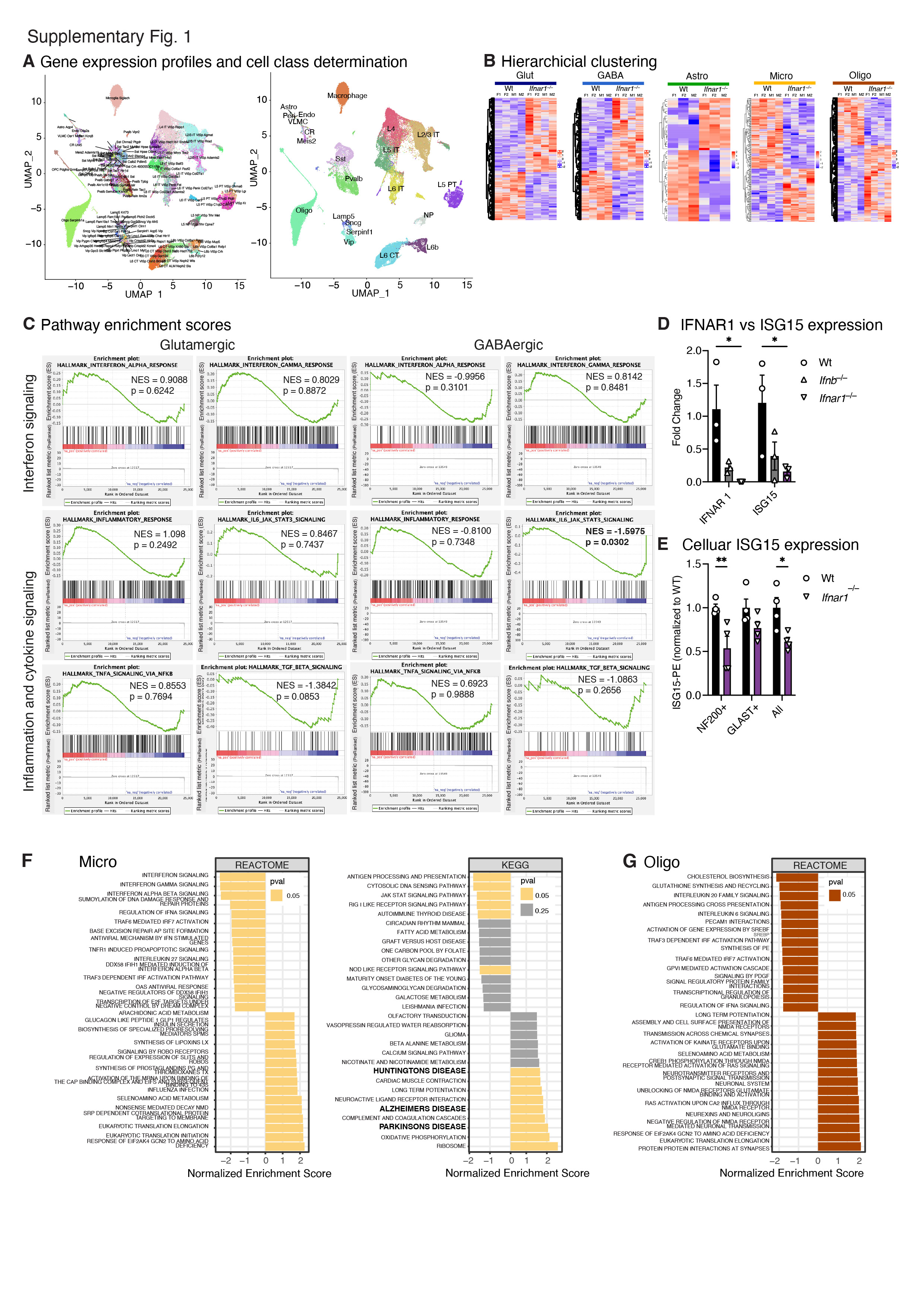

Supplement: Supplementary file 1 — Additional file1 Supplementary Fig. 1. Additional snRNA-seq analysis supplementing Fig. 2.Uniform manifold projectionsshowing complete annotations of all cell types and sub-types defining the broad cell classes used for DE analysis.Unbiased hierarchical clustering of differentially expressed genes among all 5 cortical cell classes isolated from 1.5-month-old Wt and Ifnar1−/− mice showing similarities between samples.GSEA pathway enrichment scores for pathways relating to interferon signaling, inflammation, and cytokine signaling in Glutamatergic and GABAergic cell classes. NES = Normalized Enrichment Score.Relative mRNA expression of IFNAR1 and ISG15 expression in the cortex of 3-month-old Wt, Ifnb–/–, and Ifnar1–/– miceas log2FC expression standardized to two housekeeping genesand normalized to Wt. *P < 0.05 by two-way ANOVA and Tukey’s post hoc test.Quantification of parentfrequency of ISG15-PE single cells in NF200-FITC neuronal, GLAST-APC astrocyte, and ‘All’ single cell populations analyzed by flow cytometry in 3-month-old Wt and Ifnar1–/– cortex, normalized to Wt. *P < 0.05 by two-way ANOVA and Bonferroni’s post hoc test.GSEA pathway tables showing top 15 positively and negatively enriched terms formicrogliaandoligodendrocytes. Significant neurodegenerative disease pathways are highlighted in bold text. [file 12929_2026_1257_MOESM1_ESM.jpg]

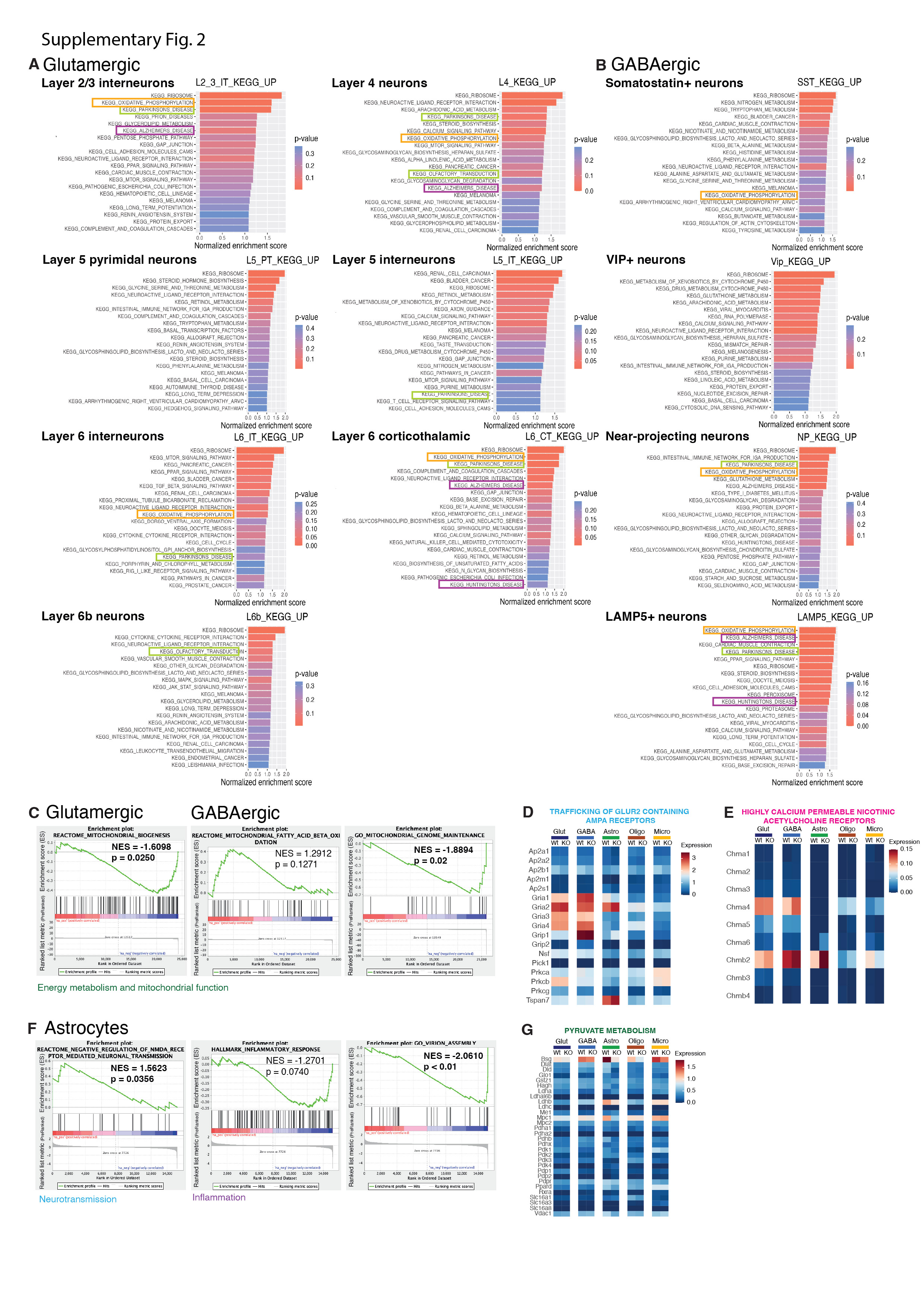

Supplement: Supplementary file 2 — Additional file2 Supplementary Fig. 2. Dysregulated GSEA pathways highlighting Parkinson’s Disease and mitochondrial metabolism pathways in Ifnar1–/– neuronal sub-types and astrocytes.Top 20 upregulated KEGG pathways corticalglutamatergic and GABAergic neuronal subtypes annotated in the snRNA-seq dataset shown in Supplementary Fig. 1B, highlighting ‘Parkinsons disease’, ‘oxidative phosphorylation’, and other neurodegenerative disease pathways such as ‘Alzheimers disease’ and ‘Huntingtons disease’.GSEA pathway enrichment scores for pathways relating to energy metabolism and mitochondrial function in cortical Ifnar1–/– vs Wt glutamatergic and GABAergic neurons. NES = normalized enrichment score.Heatmaps showing differential gene expression within the major cortical cell classes of genes within the Reactome pathways‘Trafficking of GluR2 containing AMPA receptors’ and ‘Highly calcium permeable nicotinic acetylcholine receptors’ identified as commonly affected in Ifnar1–/– glutamatergic and GABAergic neuronal classes shown in Fig. 4L and M, respectively. GSEA pathway enrichment scores for pathways relating to neurotransmission and inflammation in cortical Ifnar1–/– vs Wt astrocytes. NES = normalized enrichment score showing differential gene expression among the major cortical cell classes of genes within the Reactome pathway ‘Pyruvate metabolism’ identified in Ifnar1–/– astrocytes shown in Fig. 2N. [file 12929_2026_1257_MOESM2_ESM.jpg]

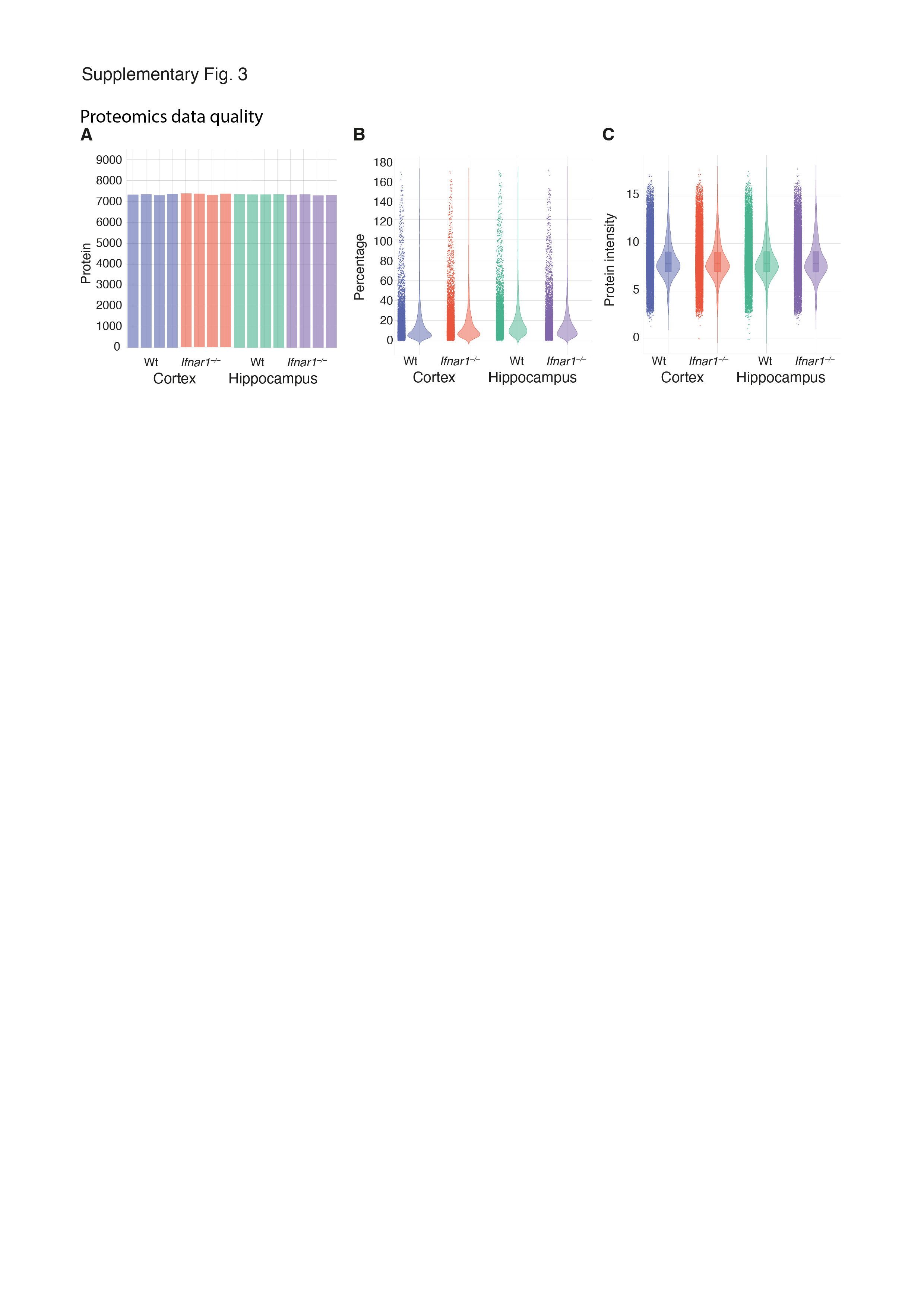

Supplement: Supplementary file 3 — Additional file3 Supplementary Fig. 3. Additional LC-MS/MS proteomics analysis supplementing Fig. 3. Quality control for proteomics analysis. Total number of proteins per sample. Coefficients of variationbetween samples. Protein intensities, showing similarity between samples. [file 12929_2026_1257_MOESM3_ESM.jpg]

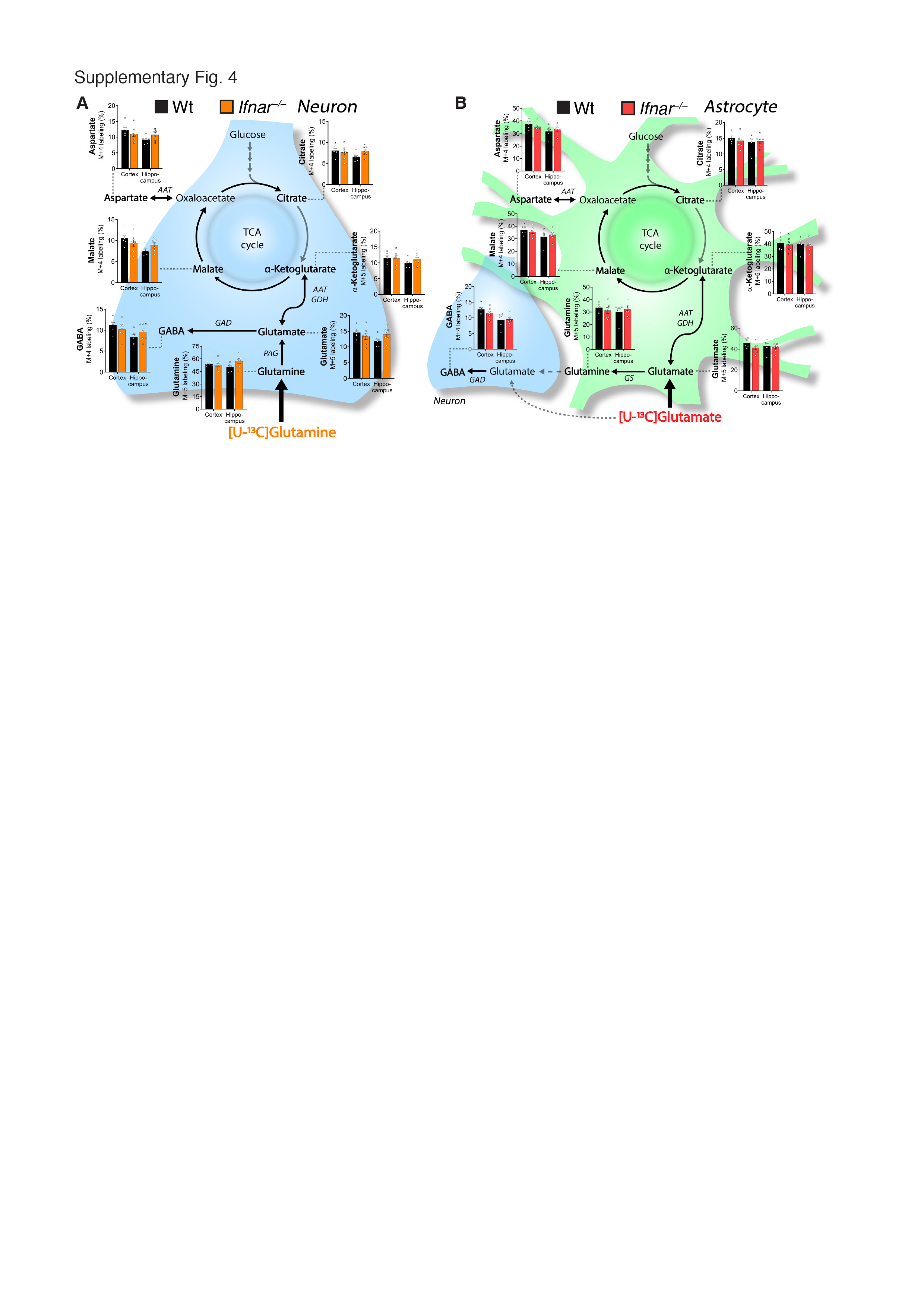

Supplement: Supplementary file 4 — Additional file4 Supplementary Fig. 4. Additional metabolic isotope labelling data supplementing Fig. 5. Cortex and hippocampal slices from 3-month-old Wt and Ifnar1−/− micewere incubated with[U-13C]glutamine or[U-13C]glutamate, which primarily reflect neuronal and astrocytic metabolism, respectively. *P < 0.05 by t-test. [file 12929_2026_1257_MOESM4_ESM.jpg]

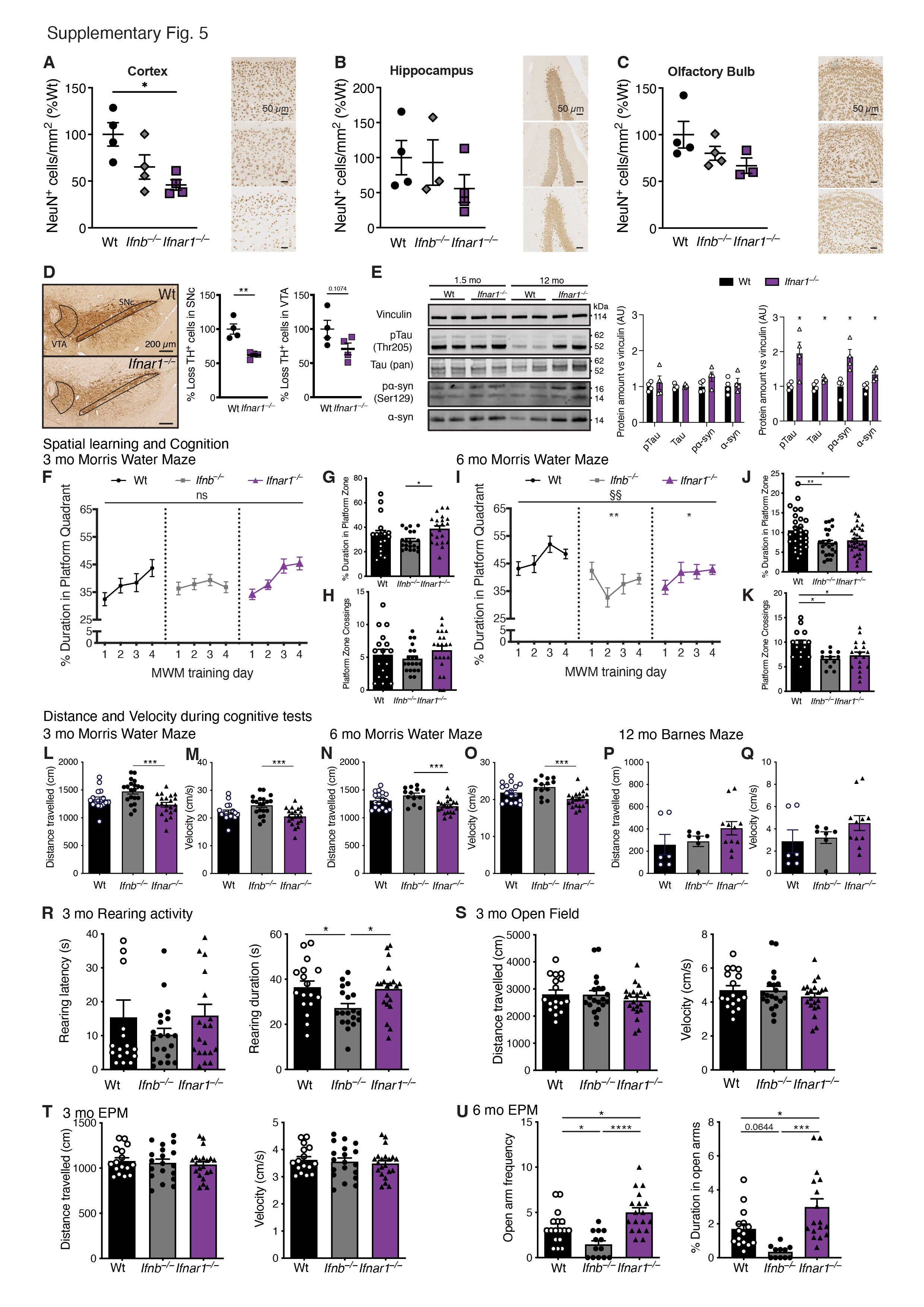

Supplement: Supplementary file 5 — Additional file5 Supplementary Fig. 5. Additional neuropathological and behavior data in Ifnar1–/–supplementing Fig. 6. Representative images and quantification of NeuN+ cells in cortex, hippocampus, and olfactory bulb of 6-month-old Wt, Ifnb–/–, and Ifnar1–/– mice as %Wt. *P < 0.05 by one-way ANOVA and Dunnett’s post hoc correction test. Representative images and quantification of % loss of TH+ cells in the substantia nigra pars compactaversus ventral tegmental area of 3-month-old Ifnar1–/– mice compared to Wt mice. **P < 0.01 by t-test. Representative immunoblots and quantifications of relative protein levels of total tau, phosphorylated tau at threonine 205, alpha-synuclein, and phosphorylated α-syn at serine 129 in brain lysates from 1.5- and 12-month-old Wt vs Ifnar1−/− mice, normalized to vinculin. *P < 0.05 by t-test. MWM results for 3-month-old and 6-month-old Wt, Ifnb−/−, and Ifnar1−/− mice. Data are mean ± SEM, n = 13-20 per genotype. MWM training performances. §§P < 0.01 for genotype effect by two-way ANOVA. Genotype effect vs Wt *P < 0.05 and **P < 0.01 by Tukey’s post hoc test. Time spent in platform zone as % duration of test session. *P < 0.05 and **P < 0.01 by one-way ANOVA and Tukey’s post hoc correction test. Number of platform zone crossings. *P < 0.05 by one-way ANOVA and Tukey’s post hoc correction test. Distanceand Velocity measurements for 3 month MWM, 6 month MWM, and 12 month Barnes maze cohorts.***P < 0.001 by one-way ANOVA and Tukey’s post hoc correction test. Additional rearing activity measurements of 3-month-old Wt and Ifnar1–/– mice, including latency to begin rearingand cumulative rearing duration. Distanceand velocity of 3-month-old Wt, Ifnb−/−, and Ifnar1−/− mice on the OF, corresponding to Fig. 6, Q.Distance and velocity of 3-month-old mice on the EPM, corresponding to Fig. 6R. EPM Open arm duration and side edge investigations of 6-month-old mice. [file 12929_2026_1257_MOESM5_ESM.jpg]

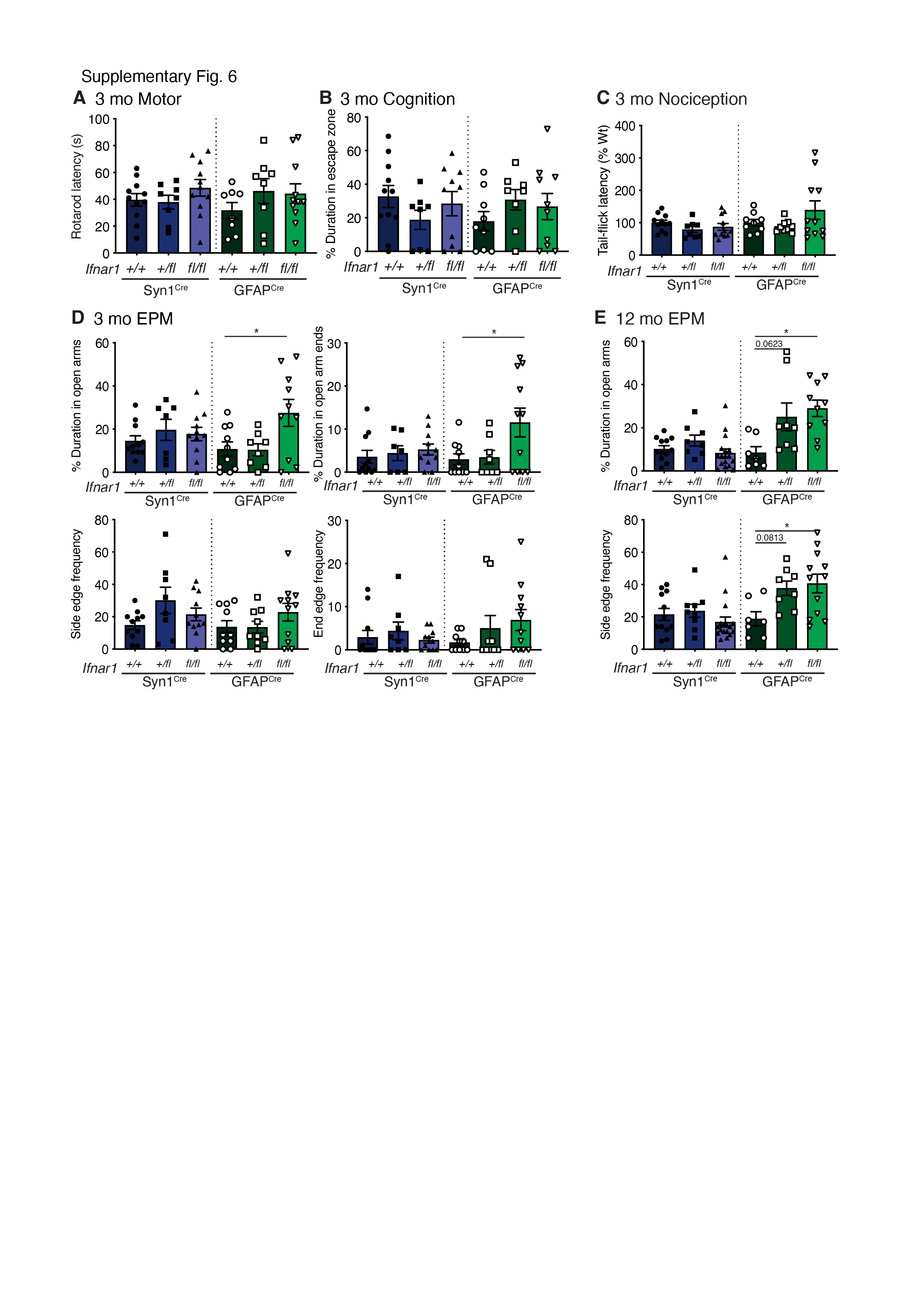

Supplement: Supplementary file 6 — Additional file 6 Supplementary Fig. 6. Additional data on conditional Ifnar1–/– strains supplementing Fig. 7. Rotarodand Barnes Maze performances of 3-month-old neuronal Syn1Cre;Ifnar1fl/fl mice and astrocytic GFAPCre;Ifnar1fl/fl mice. Nociception to heat-induced pain as %Wt performance of 3-month-old Syn1Cre;Ifnar1fl/fl and GFAPCre;Ifnar1fl/fl mice. EPM performances of 3-month-old Syn1Cre;Ifnar1fl/fl and GFAPCre;Ifnar1fl/fl mice and 12-month-old Syn1Cre;Ifnar1fl/fl and GFAPCre;Ifnar1fl/fl mice. *P < 0.05 by one-way ANOVA and Tukey’s post hoc correction test. Data in all graphs are mean ± SEM. [file 12929_2026_1257_MOESM6_ESM.jpg]
